# Molecular characterization and antimicrobial susceptibility of *Staphylococcus aureus* isolated from children with acute otitis media in Liuzhou, China

**DOI:** 10.1186/s12887-018-1366-6

**Published:** 2018-12-15

**Authors:** Yan Ling Ding, Jinjian Fu, Jichang Chen, Sheng Fu Mo, Shaolin Xu, Nan Lin, Peixu Qin, Eric McGrath

**Affiliations:** 1 Department of Laboratory, Liuzhou Maternity and Child Healthcare Hospital, Liuzhou, 545001 China; 2 Department of Neonatology, Liuzhou Maternity and Child Health Care Hospital, Liuzhou, 545001 China; 3 Department of Otolaryngology, Liuzhou Maternity and Child Health Care Hospital, Liuzhou, 545001 Guangxi China; 4 Children’s Hospital of Michigan, Detroit, MI USA; 5 Department of Pediatrics, Wayne State University School of Medicine, 3901 Beaubien Blvd, Detroit, MI 48201 USA

**Keywords:** *Staphylococcus aureus*, Acute otitis media, Antibiotic resistance, Genetic background, Pediatrics

## Abstract

**Background:**

There have been few studies focused on the prevalence, bacterial etiology, antibiotic resistance, and genetic background of *Staphylococcus aureus* (*S. aureus*) in children with acute otitis media (AOM) in China.

**Methods:**

A retrospective study was conducted in Liuzhou Maternity and Child Healthcare Hospital. Patients younger than 18 years diagnosed with AOM were enrolled in the study. Middle ear fluid specimens were collected and cultured for bacterial pathogens. The antibiotic susceptibility, virulence genes, macrolide resistant genes and sequence types of *S. aureus* were identified.

**Results:**

From January 1, 2013 to December 31, 2015, a total of 228 cases of AOM were identified. Pathogenic bacteria were positive in 181 (79.4%) of 228 specimens. *Streptococcus pneumoniae* was the most common bacteria (36.4%), followed by *S. aureus* (16.2%). Among the 37 *S. aureus* isolates, 12 (23.5%) were methicillin-resistant *S. aureus* (MRSA), and 25 (77.5%) were methicillin-susceptible *S. aureus* (MSSA). A total of 23 isolates (62.2%) were resistant to erythromycin, 40.5% of isolates were resistant to clindamycin, and 37.8% isolates were resistant to tetracycline. Twenty-three isolates were multi-drug resistant (MDR) *S. aureus*. Eighteen isolates carried the *pvl* gene. Up to 22 (59.4%) isolates expressed *ermA* gene, 8 (21.6%) isolates expressed both *ermA* and *ermC* genes, and only 8.1% expressed *ermB*. Among all *S.aureus* isolates, 7 sequence types (STs) were identified by multilocus sequence typing (MLST). The most common ST was ST59 (16/37, 43.2%), followed by ST45 (7/37, 18.9%) and ST30 (7/37, 18.9%). The predominant MSSA isolates were ST59-t437-MSSA (5/25, 20.0%), the prevailing MRSA isolates were Taiwan related strains ST59-SCCmec-IVa/V (5/12, 41.6%).

**Conclusions:**

*S. aureus* was the second most common cause for AOM in children in Liuzhou. Most of the *S. aureus* was MDR which carried a high proportion of *ermA* and *ermC* gene. CA-MRSA (ST59-SCC*mec*-I*V*/V-t437) is circulating in children with AOM. These findings support continued surveillance of *S. aureus* infections in children with AOM in both communities and hospitals.

## Background

Acute otitis media (AOM) is a common pediatric bacterial infection affecting approximately 80% of children prior to the age of 3 years [1]. The incidence of AOM in Chinese children was reported to be between 57.2 and 69.4% in children age 0–2 years [2]. AOM is the primary reason for the prescription of antibiotics in children [3]. The extensive use of antibiotics has been a public health problem in China [4]. Understanding the epidemiology and the etiology of AOM is important for the clinical selection of empiric treatment.

It was reported that the incidence of pediatric AOM and the causative pathogens varied among different regions and geographic settings. Although *Streptococcus pneumoniae* (*S. pneumoniae*), *Haemophilus influenzae* (*H. influenzae*), and *Moraxella catarrhalis* (*M. catarrhalis*) are the three leading causes of AOM in children [5], it was noted that the primary bacteria responsible for AOM in Chinese children are *S. pneumoniae*, *Staphylococcus aureus* (*S. aureus*) and *H. influenzae* [2]. *S. aureus* was considered a major pathogen that led to infection and hospitalization in pediatric patients, including healthy subjects in the community in past decades [6, 7]. Although methicillin-resistant *Staphylococcus aureus* (MRSA) causing pediatric infections such as skin and soft tissue infections, pneumonia, and blood stream infections are well documented, detailed studies of the contribution of *S.aureus* (both MRSA and methicillin-sensitive *Staphylococcus aureus*, MSSA) to AOM are limited. There have been few studies focused on the epidemiology of pediatric AOM in China. The aim of this study was to both evaluate the bacterial etiology of AOM and the antibiotic resistance patterns of *S. aureus* in pediatric AOM disease and investigate the molecular features and genetic background of *S. aureus* AOM in children from western China.

## Methods

### Patients and sample collections

This retrospective study was conducted between January 1, 2013 and December 31, 2015 in the otolaryngology clinic of Liuzhou Maternity and Child Healthcare Hospital. Patients younger than 18 years were enrolled in the study. The diagnostic criteria for AOM was based on the International Classification of Diseases, ninth version, Clinical Modification (ICD-9-CM) code 3810, 3820, or 3829 [3]. Any child diagnosed with chronic otitis media, or who had prior history of tympanostomy tube insertion, cholesteatomas, or otitis externa were excluded. Spontaneous ear pus drainage from the deep ear canal was swabbed by otolaryngologists and then sent to the microbiology laboratory.

The specimens were immediately plated on Columbia agar containing 5% sheep blood, on chocolate agar and on MacConkey agar. All agars were placed in 35–37 °C, 5–10% CO_2_ incubated for 24 h to 48 h. The suspected bacteria were identified using VITEK 2 compact automatic microbial analysis system (Biomérieux, Marcyl’ Etoile, France).

### Antimicrobial susceptibility test

Antimicrobial susceptibility test of *S. aureus* was performed using the Gram-positive cocci antibiotic cards (Biomérieux, Marcyl’ Etoile, France). Minimum inhibitory concentrations (MICs) were proposed using in-house prepared panels according to Clinical and Laboratory Standards Institute (CLSI) guidelines [8]. Isolates not susceptible to at least 3 different antibiotic classes such as β-lactams, macrolides, and glycopeptides were defined as multidrug-resistant (MDR) *S. aureus.*

### Detection of the *mecA*, *Panton-Valentine Leukocidin* (*pvl*) and erythromycin-resistance genes

The *mecA* and *lukS-PV or lukF-PV* genes (both of which encode for *pvl*) were detected as described previously [9]. The macrolide resistance genes *ermA, ermB* and *ermC* were amplified by PCR methods for all erythromycin-resistance isolates [10].

### SCCmec typing

The staphylococcal cassette chromosome *mec* (SCC*mec*) was distinguished by the updated multiplex PCR assay developed by Zhang K et al. [11].

### Multilocus sequence typing (MLST)

MLST was performed by PCR amplification and sequencing of 7 housekeeping genes by using primers and protocols described previously [12]. DNA sequences were submitted to the MLST database website (www.mlst.net) for the generation of an allelic profile and sequence type (ST).

### Spa typing

*Spa* typing was determined by using established method [13]. Sequences were submitted to the RIDOM web server (http://spaserver.ridom.de) for assignment of the *spa* type.

### Statistical analysis

Data were analyzed using descriptive statistics and χ^2^ tests. The two-sided *p*-value for statistical significance was defined as *p* < 0.05. All statistical analyses were conducted using SPSS version 20.0 (SPSS Inc. Chicago, Il, USA).

## Results

### Epidemiology and microbiology

Two hundred and twenty-eight children age 0–15 years were identified with AOM in the otolaryngology clinic during the study period. The median age was 24 months. Sixty-six percent of them were less than 2 years. The male-to-female ratio was 1:0.6. (Table 1). Pathogenic bacteria were positive in 181 (79.4%) of 228 specimens, *S. pneumoniae* was the most common bacteria (36.4%), followed by *S. aureus* (16.2%), *Pseudomonas aeruginosa* (4.4%) and *H. influenzae* (3.9%) (Table 2).

**Table 1:** The demographic information of children with AOM

Characteristic	AOM		Staphylococcus aureus positive	
	N	%	N	%
Gender				
Male	141	61.8	21	56.8
Female	87	38.2	14	43.2
Age (years)				
< 1	106	46.5	17	45.9
1-	46	20.2	8	21.6
2-	49	21.5	7	18.9
≥5	27	11.8	5	13.5

**Table 2:** Microbiology of middle ear fluid from children with acute otitis media

Pathogen	No. of strains (%)
No growth	47 (20.6)
Any growth	181 (79.4)
Streptococcus pneumoniae	83 (36.4)
*Staphylococcus aureus*	37 (16.2)
Haemophilus influenzae	9 (3.9)
Streptococcus pyogenes	4 (1.7)
Moraxella catarrhalis	1 (0.4)
*Candida albicans*	5 (2.2)
Fungus	6 (2.6)
Pseudomonas aeruginosa	10 (4.4)
*Klebsiella pneumoniae*	4 (1.7)
*Escherichia coli*	3 (1.3)
Proteus mirabilis	2 (0.8)

Among the 37 *S. aureus* isolates, 12 (23.5%) were MRSA, and 25 (77.5%) were MSSA. All isolates were susceptible to ciprofloxacin, rifampicin, linezolid and vancomycin. A total of 23 isolates (62.2%) were resistant to erythromycin, and 37.8% isolates were resistant to tetracycline. The resistant rate to clindamycin was higher in the MSSA group than in the MRSA group (*p* = 0.040) (Table 3). Twenty-three isolates were multi-drug resistant (MDR) *S. aureus*. In the MRSA group, the MDR rate was 83.3%, while in the MSSA group, the MDR rate was 52.0%. The most common MDR pattern was resistance to penicillin/erythromycin /clindamycin/tetracycline.

**Table 3:** Antimicrobial susceptibilities of *Staphylococcus aureus* isolated from children with AOM

Antibiotic	Susceptibilities rate (%)			*P* value
	Overall (*n* = 37)	MSSA (*n* = 25)	MRSA (*n* = 12)	
Penicillin	4 (10.8)	4 (16.0)	0 (0.0)	0.282
Gentamicin	35 (94.6)	23 (92.0)	12 (100.0)	1.000
Erythromycin	14 (37.8)	12 (48.0)	2 (16.7)	0.066
Tetracycline	23 (62.2)	16 (64.0)	7 (58.3)	0.739
Ciprofloxacin	37 (100.0)	25 (100.0)	12 (100.0)	1.000
Clindamycin	15 (40.5)	13 (52.0)	2 (16.7)	0.040
Sulfamethoxazole- trimethoprim	36 (97.3)	24 (96.0)	12 (100.0)	1.000
Chloramphenicol	36 (97.3)	25 (100.0)	11 (91.7)	1.000
Rifampicin	37 (100.0)	25 (100.0)	12 (100.0)	1.000
Linezolid	37 (100.0)	25 (100.0)	12 (100.0)	1.000
Vancomycin	37 (100.0)	25 (100.0)	12 (100.0)	1.000

### Virulence and macrolide-resistance genes

Eighteen *S. aureus* isolates carried the *pvl* gene. The *pvl* gene distribution varied between the MRSA and the MSSA groups, with 9 MRSA isolates (75.0%) and 9 MSSA isolates (36.0%) carring the *pvl* gene, with MRSA isolates having a higher proportion than the MSSA group (χ^2^ = 4.94, *p* = 0.026). Up to 22 (59.4%) isolates expressed the *ermA* gene, and 8 (21.6%) isolates expressed both *ermA* and *ermC* genes, and only 8.1% expressed *ermB*. Eighty-three and 41 % of MRSA isolates expressed *ermA* and *ermC* genes, respectively, while only 12 (32.4%) and 4 (10.8%) of MSSA isolates expressed *ermA* and *ermC* gene, which was significantly different (*p* = 0.016, and 0.002, respectively) (Table 4).

**Table 4:** Prevalence of erythromycin resistant genes among Staphylococcus aureus isolated from children with AOM

Gene	No. of positive isolates (%)	No. distributing in		*P* value
		MSSA (n = 25)	MRSA (n = 12)	
ermA	22 (59.4)	12 (32.4)	10 (83.3)	0.002
ermB	3 (8.1)	1 (2.7)	2 (16.7)	0.144
ermC	9 (24.3)	4 (10.8)	5 (41.7)	0.016
ermA+ermC	8 (16.3)	4 (10.8)	4 (33.3)	0.067
ermA+ermB	2 (5.4)	0 (0.0)	2 (16.7)	1.000
ermB+ermC	1 (2.7)	0 (0.0)	1 (8.3)	1.000
ermA+ermB+ermC	1 (2.7)	0 (0.0)	1 (8.3)	1.000

### Molecular typing

Among the 12 MRSA isolates, 4 (33.3%) belonged to SCC*mec* type IVa, 5 (41.6%) belonged to SCC*mec* type IV, and 3 (25.8%) belonged to SCC*mec* type V. Twelve *Spa* types were identified, t437 (13/37, 35.1%) was the most common type, followed by t037 (6/37, 16.2%), and t021 (4/37, 10.8%). The t437 (8/12, 75.0%) and t437 (5/25, 20.0%) type was the most common *Spa* type in the MRSA and the MSSA groups, respectively.

**Fig. 1:**
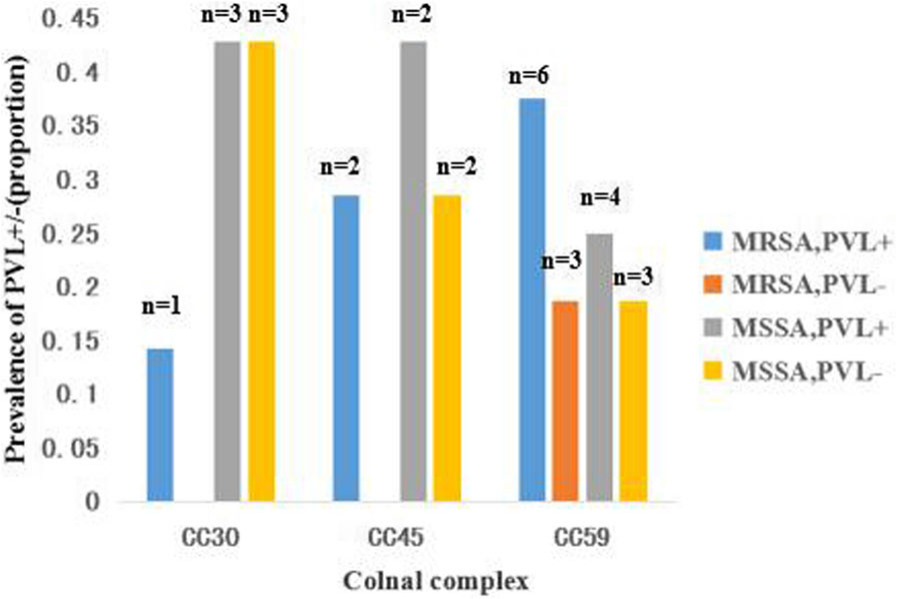
The *pvl* gene distribution among *S. aureus* isolates

Among all *S. aureus* isolates, 7 sequence types (STs) were identified by MLST. The most common ST was ST59 (16/37, 43.2%), followed by ST45 (7/37, 18.9%) and ST30 (7/37, 18.9%) (Table 5). The predominant MSSA isolates were ST59-t437-MSSA (5/25, 20.0%), the second predominant MSSA were southwest-pacific strains ST30-t037-MSSA (4/25, 16.0%). The prevailing MRSA isolates were Taiwan related strains ST59-SCC*mec*-IVa/V (5/12, 41.6%), most of them were found among children older than 2 years (4/5, 80.0%). The Berlin strains ST45-SCC*mec*- IVa/V (2/12, 16.7%) were found in 2 infants aged 3 months. In the ST59-SCC*mec*-IV/IVa/V-t437 clone, the antibiotic resistant profile was erythromycin/clindamycin /tetracycline. Moreover, the ST59-SCC*mec*-I*V*/V-t437 clone showed high resistance to erythromycin, clindamycin, and tetracycline, which was 88.9, 88.9, and 44.5%, respectively. Additionally, ST59 was the most frequent ST in *pvl* positive isolates, including 2 SCC*mec* type IV, 2 SCC*mec* type IVa, and 2 SCC*mec* type V. Other STs found in *pvl* positive isolates included ST30 (3 MSSA, 1 MRSA), ST45 (2 MSSA, 2 MRSA), and ST59 MSSA (4 isolates). Figure 1 showed the *pvl* gene distribution among the CC30, CC45 and CC59 strains, with a high proportion of *pvl* gene distribution in CC59 strains.

**Table 5:** Molecular characteristics and antibiotic resistance profiles of *Staphylococcus aureus* isolated from children with AOM

Variables	MRSA (n = 12)	MSSA (n = 25)
SCC*mec* (n)	IV (5), IVa(4), V(3)	–
CCs (n)	CC30(1),CC45(2),CC59(9)	CC30(6),CC188(1), CC45(5),CC59(7), CC88(1), CC942(2)
STs(n)	ST30(1),ST45(2),ST59(9)	ST188(13),ST30(6), ST398(1),ST45(5), ST59(7), ST88(1),ST942(2)
*Spa* (n)	t037(1),t0181(2),t3845(1),t437(8)	t021(4),t037(5),t1081(1),t1445(32), t189(3),t2592(1),t3551(1), t3590(1),t3736(1),t437(5),t571(1)
*pvl*(n)	9	9
erm-resistant genes (n)	*ermA*(10).*ermC*(5),*ermB*(2)	*ermA*(2).*ermC*(4),*ermB*(1)
Antibiotir resistance profiles (n)	P(12),E(10),DA(10),Cl(1),TE(5)	P(21),SXT(1),GN(2),E(13),DA(12),TE(9)
MLSBi (n)	3	4

*P* penicillin, *E* erythromycin, *GN* gentamicin, *TE* tetracycline, *DA* clindamycin, *Cl* chloramphenicol, *SXT* Sulfamethoxazole- trimethoprim

## Discussion

AOM is a disease with worldwide prevalence having broad disease burden and may require prolonged treatment courses because at least a third of children have two or more episodes of AOM (recurrent AOM) in the first three years of life [14]. Reliable epidemiological data on etiology and burden of AOM are important as the data help clinicians with the selection of appropriate empiric antibiotic therapy for pediatric AOM and for public health policy decision-making.

In this retrospective study, we found that *S. pneumoniae* and *S. aureus* were the most predominant etiologic agents causing AOM, being isolated in 36.4 and 16.2% of the specimens of children with AOM, respectively. Most of the *S. aureus* was MDR and resistant to erythromycin, clindamycin and tetracycline. The first two antibiotics (erythromycin and clindamycin) were the most frequent medicines prescribed by Chinese pediatricians for infectious diseases [4]. Historically, the major bacteria responsible for most cases of AOM were *S. pneumoniae* and *H. influenzae* [15]. The etiology of the pathogenic bacteria does not appear to have changed significantly over time. Since the prevalence and the main causal agents of AOM varied by geographic location, we observed a different epidemiology and etiology from previous studies [1, 3, 5] which revealed that the most causal agents of AOM were *S. pneumoniae* and *H. influenzae*, but our study was in line with one study conducted in southern China which demonstrated that the major pathogens causing AOM were *S. pneumoniae* and *S. aureus*, which accounted for 47.2 and 18.5% of the specimens isolated from AOM patients, respectively [2]. It has been reported that in the era of universal pneumococcal conjugate vaccine (PCV) immunization, that *H. influenzae* may become the predominant pathogen of AOM, suggesting that the introduction of PCV7 can change the relative prevalence of main causal agents [16]. The same result was observed in Saudi children, after the introduction of pneumococcal vaccines in the routine immunization schedule, *S. aureus* has become the most predominant contributor to AOM [17]. The determinants of why *S. aureus* has become the second most common causal agent of AOM in China is poorly understood. In China, as the *H. influenza* b vaccine and PCV were self-paid and did not enter into the Chinese Expanded Program on Immunizations (EPI), we didn’t see the changes of pathogen patterns distributed in the AOM disease for the vaccination of *H. influenza* b vaccine and PCV. The low coverage of PCV7 and antibiotic overuse and abuse in China can partly explain this disparity [2]. In this region of the world, *S. aureus* should be considered and targeted with appropriate therapy if initial therapies targeting *S. pneumoniae* fail to lead to clinical improvement, especially if culture is not available.

Antibiotic resistance has become an important public health problem in mainland China. Restriction of β-lactam use in MRSA infections required use of other types of antibiotic options for treatment. However, except for resistance to all kind of β-lactam antibiotics, the MRSA isolates found in our study developed a high resistant rate to non-β-lactam antibiotics, especially to erythromycin, clindamycin and tetracycline. We found that the resistance rate to clindamycin in MSSA is even higher than in MRSA isolates. It was reported that both erythromycin and clindamycin have been common prescribed antibiotics for *S. aureus* infection [18]. A high resistance rate was also reported in mainland China [19], which indicated that the high antibiotic resistance rate of *S. aureus* is a common public health problem in China and that the two antibiotics were not the priority options for the empiric antibiotic therapy in pediatric infections. It was previously reported that in the macrolide resistance isolates, there were 59.4, 24.3, and 21.6% of which carried *ermA*, *ermC* and both *ermA* and *ermC* gene, respectively. Our study was consistent with a previous report that showed that of resistant *S. aureus* isolates, 37.7% had *ermA*, 26.6% had *ermC* and 18.6% had both *ermA* and *ermC* genes [20], but different from a study conducted in Turkey which showed that 50% of *ermA* positive isolates also carried the *ermC* gene [10].

As a pathogen with extremely high prevalence, *S. aureus* causes various clinical infections such as skin and soft tissue infections (SSTIs) [19], invasive community-acquired MRSA (CA-MRSA) infections [21], and pneumonia [22]. Few studies about MRSA and MSSA isolates contemporaneously circulating among age-specific groups of children attending otolaryngology clinics have been examined [23, 24]. According to the previous report [11], hospital-acquired MRSA (HA-MRSA) is usually detected with SCC*mec* type I, II and III, while CA-MRSA is usually detected with SCC*mec* type IV, IVa and V. In this study, all of the MRSA isolates carried SCC*mec* IV, IVa and V, which confirmed that these MRSA isolates belonged to CA-MRSA. Twelve *Spa* types and seven ST types clustered into 7 clonal complex (CCs) among MSSA and 3 CCs among MRSA were observed in our study, indicating that there is great genetic diversity in *S. aureus* isolated from AOM patients. MSSA isolates with a genetic background (ST30-*t*037, ST45-*t*1081 and ST59-*t*437) was common to MRSA clones in this study suggesting that these MSSA isolates might have the potential to become CA-MRSA clones once acquisition of the *mecA* gene occurs [21].

Despite the high prevalence, only a few epidemic clones have been identified in China [25–27]. Previous studies throughout mainland China found that ST59- SCC*mec*-IVa/V strains were the most common strains causing CA-MRSA infections among children [25–28]. Our study also confirmed that the predominant sequence type of MRSA isolated from AOM children was ST59, which accounted for 75% of all the MRSA isolates. The previous report of ST59 was detected from a few MSSA isolates and in a single MRSA isolate in the United States, a large proportion of ST59 emerging in Taiwan was reported in 2004 and ST59-MRSA was called Taiwan clone [29]. ST59 was not only predominant in Shanghai [30], Guangzhou [31], and Taiwan [29], but also served as prevailing strains in Hongkong [32] and Vietnam [33]. The Asian Network for Surveillance of Resistant Pathogens (ANSORP) study conducted in 17 hospitals from Asian countries demonstrated that the predominant clones of CA-MRSA isolates were ST59-MRSA-SCCmec type IV-spa type t437 [34]. These findings suggested that ST59 is currently spreading between adjacent regions and supporting its dominance in the Asian region as a whole [33]. It is widely assumed that the CA-MSSA isolates acquiring the resistance gene *mecA* would become the major sources of CA-MRSA. In our study, we observed that ST59-MSSA was the predominant sequence type in the MSSA group, accounting for 28% of all MSSA isolates, which indicated that the *S. aureus* isolates undergoing genetic variations have great capacities for environmental adaption. The similar genetic background of ST59 between MRSA and MSSA isolates was also observed in ST30 and ST45 in our study.

ST45 was the second prevailing ST in our study, accounting for 20% of MSSA and 16.7% of MRSA isolates. It was reported that clonal complex 45 (CC45) is common throughout European countries such as Germany and the Netherlands and Belgium [35]. The ST45-SCC*mec*-I*V*/V-t437 clone is well known as the Berlin clone. The Berlin clone was first observed in 1993, and its emergence was attributed to acquisition of *mecA* by a community clone of MSSA [36]. The ST45 now spread in Hongkong [35] and mainland China [37], including in western China where our study was conducted. It was speculated that CC45 strains may be more transmissible among health care settings and hospitals [35].

One of the interesting findings was that ST398-MSSA was found in this study. ST398 is considered as a livestock-associated pathogen mainly affecting people in contact with major animal reservoirs [38]. It is noteworthy that this AOM case with isolates of ST398 reported no direct livestock-associated risk factors, although many reports documented that persons living in places of high livestock density were found to have a greater chance of livestock-associated CC398 carriage even if they lacked direct contact with animals [39, 40]. CC398 may now be sporadic and distributed in China including areas such as Shanghai [30] and Liuzhou. This study finding suggests the probability of CC398 transmission via human contact instead of animal contact [41].

Panton-Valentine leukocidin (PVL) is a bicomponent toxin that can cause the lysis of leucocytes and it is a main virulence factor of *S. aureus* which is responsible for severe invasive disease such as necrotizing pneumonia [30]. An important finding in this study was the high detection rate of the *pvl* gene in *S. aureus* isolates, with significant differences between the MRSA and the MSSA groups. Our result was consistent with previous reports indicating that the *pvl* gene was more common in MRSA isolates than in MSSA isolates [42]. Several studies found that the proportion of *pvl* positivity was approximately 27–40% among *S.aureus* isolates detected from children in mainland China [30]. In the current study, the *pvl* gene was found in ST30, ST45 and ST59 clones. It was reported that CA-MRSA ST59 isolates had significantly more pronounced virulence than the geographically matched HA-MRSA clones ST239 in various animal models, including the *pvl* gene [43]. The CC59 was predominant among *pvl* positive CA-MRSA in mainland China [30], for example, Li et al. [44] reported 55.5% of CC59 MRSA isolates to be *pvl* positive in China, while we detected 66.7% of CC59 MRSA isolates with *pvl* positive in AOM disease.

There are some limitations to our study. First of all, the single-center design and the small number of AOM patients may limit the generalizability of our study results. Secondly, the AOM cases in this study may not accurately represent all AOM cases as we swabbed spontaneous ear pus drainage from the deep ear canal and the external auditory canal to culture organisms, the results of which may or may not have included the true middle ear pathogen. *S.aureus* may have been a leading cause of AOM, but as we swabbed the ear canal, this may lead to detection of some colonizing agents such as *S.aureus*. Lastly, a retrospective review of medical records for identifying patients presented to an Otolaryngology clinic may have potentially decreased the generalizability of the results, as some children may have had more severe disease which were referred to a surgeon, as opposed to a primary care provider.

## Conclusion

In conclusion, *S. aureus* was a leading cause for AOM in children in Liuzhou. Most of the *S. aureus* was MDR and carried high proportion of *ermA* and *ermC* gene. CA-MRSA (ST59-SCC*mec*-I*V*/V-t437) is circulating in children with AOM, suggesting a potential for CA-MRSA transmission from community to hospital. These findings support growing concern about continued surveillance of *S. aureus* infections in both communities and hospitals, and raise questions about the routine antibiotic use for the treatment of *S. aureus* infections in China and in countries worldwide.

## Abbreviations

ANSORP: Asian Network for Surveillance of Resistant Pathogens
AOM: Acute otitis media
CA-MRSA: Community-acquired MRSA
CCs: Clonal complexs
CLSI: Clinical and Laboratory Standards Institute
EPI: Expanded Program on Immunization
*H. influenzae*: *Haemophilus influenzae*
HA-MRSA: hospital-acquired MRSA
*M. catarrhalis*: *Moraxella catarrhalis*
MDR: Multi-drug resistant
MICs: Minimum inhibitory concentrations;
MLST: Multilocus Sequence Typing
MRSA: Methicillin- resistant *S.aureus*
MSSA: Methicillin-susceptible *S.aureus*
PCV: Pneumococcal conjugate vaccine
*pvl*: *Panton-Valentine Leukocidin*
*S. pneumoniae*: *Streptococcus pneumoniae*
*S.aureus*: *Staphylococcus aureus*
SCC*mec*: Staphylococcal cassette chromosome *mec*
SSTIs: Skin and soft tissue infections
STs: Sequence types
